# Toxicological Study and Genetic Basis of BTEX Susceptibility in *Drosophila melanogaster*

**DOI:** 10.3389/fgene.2020.594179

**Published:** 2020-10-15

**Authors:** Temitope H. Adebambo, Donald T. Fox, Adebayo A. Otitoloju

**Affiliations:** ^1^Department of Pharmacology and Cancer Biology, Duke University School of Medicine, Durham, NC, United States; ^2^Department of Zoology, University of Lagos, Lagos, Nigeria

**Keywords:** BTEX, *Drosophila melanogaster*, apoptosis, development, imaginal disk, GWAS, DGRP

## Abstract

Benzene, toluene, ethylbenzene and xylene, also known as BTEX, are released into environmental media by petroleum product exploratory and exploitative activities and are harmful to humans and animals. Testing the effects of these chemicals on a significantly large scale requires an inexpensive, rapidly developing model organism such as *Drosophila melanogaster*. In this study, the toxicological profile of benzene, toluene, ethylbenzene, p-xylene, m-xylene, and o-xylene in *D. melanogaster* was evaluated. Adult animals were monitored for acute toxicity effects. Similarly, first instar larvae reared separately on the same compounds were monitored for the ability to develop into adult flies (eclosion). Further, the impact of fixed concentrations of benzene and xylene on apoptosis and mitosis were investigated in adult progenitor tissues found in third instar larvae. Toluene is the most toxic to adult flies with an LC_50_ of 0.166 mM, while a significant and dose-dependent decrease in fly eclosion was observed with benzene, p-xylene, and o-xylene. An increase in apoptosis and mitosis was also observed in animals exposed to benzene and p-xylene. Through Genome Wide Association Screening (GWAS), 38 regions of the *D. melanogaster* genome were identified as critical for responses to p-xylene. This study reveals the strength of *D. Melanogaster* genetics as an accessible approach to study BTEX compounds.

## Introduction

Volatile organic aromatic compounds (mainly BTEX) have experienced a surge in their environmental presence as a result of increased industrial combustions, vehicular movement, and petroleum operations (Ojiodu, 2013; Cheng et al., 2018). Several years of oil exploration by Multinational Corporations as well as the spillage and gas flaring associated with such activities has led to a degraded environment (Ebegbulem et al., 2013). Release of petroleum hydrocarbons into the environment, whether by accident or due to anthropogenic activities, impacts water and soil and may contribute to regional or atmospheric pollution (Ite et al., 2013). Aside from oil spills and accidents, petroleum pollutant sources could be due to lack of maintenance culture, deliberate acts of violence, bunkering evaporative emission in gas stations, and release of volatile components of gasoline through automobile exhausts (McInnes, 1996; Nwilo and Badejo, 2005). Benzene, toluene, ethylbenzene, and xylene (BTEX) and other aromatic hydrocarbons are predominant components of emissions from gasoline and diesel-powered engines (Friedrich and Obermeier, 1999). Leading producers of petroleum products are now characterized by large scale oil utilization and industrial processes, leading to high pollution and release of volatile organic compounds (Olumayede and Okuo, 2012). Benzene and its alkylated derivatives are well-known for their mutagenicity and carcinogenicity in living organisms and are threat to public health. The United States Environmental Protection Agency (EPA) has classified benzene as a group A carcinogen (Snyder and Hedli, 1996; Thammakhet et al., 2004). Xylene in particular has been recommended to be listed in the National Priorities List (NPL) by the EPA (HHS, 2007).

Occupational exposures to BTEX occur at work places via inhalation or adsorption through the skin. Exposure levels to these compounds at the industrial level have been established through many monitoring and risk assessment studies (Moolla et al., 2015; Ruchirawat et al., 2010; Ciarrocca et al., 2012). Workers and drivers at refueling stations are exposed to high concentrations of BTEX in vehicles and in gas station environments (Esteve-Turrillas et al., 2007; Hazrati et al., 2016; Mosaddegh Mehrjerdi et al., 2014). Histopathological technicians are a high-risk group because they constantly come in contact with solvents already contaminated with xylene (Kandyala et al., 2010).

Benzene, toluene, ethylbenzene, and xylene compounds are not exclusively important in industrial areas. These compounds also pose a threat to household environments as samples of BTEX have been discovered in residential areas and other locations distant from gas stations and other major sources (Correa et al., 2012; Liu et al., 2013). Deliberate inhalation of paint or glue by solvent abusers may lead to a high level of exposure to toluene and other chemicals (Dorsey, 2000). Toluene exposure values at newsstands and at home during the use of magazines adds to the total human health impact originally produced during production and retail stages (Walser et al., 2013). Exposure to these toxicants presents health effects at the organismal level that are due to direct or indirect impact on the DNA at the subcellular level (Mandani et al., 2013; Shaikh et al., 2018; Warden et al., 2018). Experiments conducted with earthworms and *Drosophila melanogaster* indicate a relationship between BTEX exposure and DNA damage/fragmentation (Liu et al., 2010; Singh et al., 2011). Researchers have also linked neurotoxicity and loss of reflexes in factory workers to exposure to certain concentrations of benzene (Mandiracioglu et al., 2011). Pumo et al. (2006) reported behavioral, motor, and cognitive changes in the neonates of pregnant animals acutely exposed to benzene. In rats, toluene, ethylbenzene and the three isomers of xylene caused reduced body weights at birth and developmental delay (Thiel and Chahoud, 1997; Saillenfait et al., 2003). There is also an association between benzene exposure and the presence of micronuclei as well as other forms of DNA damage (Kim et al., 2010; Bucker et al., 2012; Salem et al., 2018). Toluene exposure is linked to induction of repression of genes in pathways associated with synaptic plasticity and mitochondria function (Hester et al., 2011). Altered expression of genes associated with stress has been reported with benzene, toluene and xylene exposure in *D. melanogaster* (Singh et al., 2009, 2010). Benzene exposure in adult flies also resulted in upregulation of transporter proteins involved in the sequestration of the conjugation product of the phase II detoxification reaction (Sharma et al., 2018).

One of the major challenges to advancement in the field of toxicology is to be able to correlate variation at the molecular level with phenotypic variation in quantitative traits such as susceptibility to disease and resistance to toxins (Mackay et al., 2012; Mackay and Huang, 2018). Genome Wide Association Studies (GWAS) can serve as an effective means to pull putative information from Single Nucleotide Polymorphisms (SNPs) to further learn about certain genes and the influence they have on phenotypic expression (Weeger, 2018).

This study utilizes the exposure of a model organism, *D. melanogaster*, to assess effects on both animal development and acute toxicity (in adults) of BTEX environmental pollutants. *D. melanogaster*, also known as the fruit or vinegar fly, is a reliable model organism due to its rapid life cycle and wide array of genetic reagents that are available (Gilbert, 2008; Pandey and Nichols, 2011; Ruden et al., 2012). Adult *D. melanogaster* contain numerous organs that are functionally equivalent to mammalian organs, including a heart, a respiratory organ system (trachea), and a gut (Rothenfluh and Heberlein, 2002). This study also takes advantage of the *Drosophila* Genetic Resource Panel (DGRP), consisting of 200 wild-derived inbred fly strains. This collection is a powerful community resource for interrogating heritable, natural variation and linking complex traits to underlying genotypes (Huang et al., 2014). The panel presents an excellent genetic model system for quantitative genetic analyses of complex traits, and has resulted in the identification of genetic networks that underlie several stress responses, such as starvation resistance, chill coma recovery, startle behavior, oxidative stress sensitivity, radiation resistance, and exposure to alcohol (Morgan and Mackay, 2006; Jordan et al., 2012; Vaisnav et al., 2014; Weber et al., 2012). Our goal here was to determine the acute, genotoxic and developmental effect of BTEX compounds on *D. melanogaster*, and to identify genetic regions that may impact BTEX susceptibility.

## Materials and Methods

### Test Chemicals

The test chemicals benzene, toluene, ethylbenzene and xylene (p-xylene, m-xylene, and o-xylene) were supplied by VWR (us.vwr.com). Different concentrations of test chemicals were prepared using serial dilution in dimethyl sulfoxide (DMSO). Absolute ethanol ≥99.5% and glacial acetic acid (Millipore Sigma) were used in the preparation of grape juice agar.

### *Drosophila melanogaster* Culturing

The *D. melanogaster* strains used in most experiments were *white*^1118^ (*w*^1118^) with the exception of the GWAS study, which used the DGRP strains. The flies were reared in fly vials (diameter 25 mm × 90 mm) at a constant temperature of 25 ± 1°C in darkness except when they were transferred to a fresh medium. The flies were raised under a standard *D. melanogaster* media using molasses with other recipes as prepared by Archon Scientific; Durham, NC, United States^1^.

### Adult Acute Exposure Studies

*w*^1118^ adult flies, aged 0–3 days old, were maintained in standard *D. melanogaster* food. Ten flies were placed in each replicate vial containing *D. melanogaster* molasses-based food (*n* = 10 per trial, 2 trials total). The flies were treated at different concentrations obtained from range finding experiments of benzene (0, 0.11, 0.23, 0.34, 0.45, 0.56, and 1.13 mM), toluene (0, 0.11, 0.16, 0.24, 0.33, and 0.65 mM), p-xylene, m-xylene (0, 0.11, 0.16, 0.27, 0.32, and 0.65 mM), and 0.3% DMSO (Nazir et al., 2003). The BTEX compounds were mixed into the food which was melted using a microwave. The flies were exposed to the compounds for 4 days and the mortality of flies was recorded on each day during that time period. The flies were fed in new vials containing fresh concentrations of the test compounds daily. The experiment was conducted at 25°C, eliminating heat stress as a potential confounder.

### Larval Development Studies

The developmental toxicity experiment was conducted using a grape juice gel agar recipe developed by Salvaterra et al. (2006) and was modified in this study using standardized proportions for the laboratory. A liter of grape juice agar was prepared using 250 ml 100% frozen grape juice, 18 g agar, 12 ml acetic acid, 750 ml distilled water, and 12 ml 95% ethanol.

A population of flies containing 20 females and 10 males collected under light CO_2_ anesthesia were placed in standard egg collection cages covered with grape juice agar petri-dishes (Durham et al., 2014). The grape juice gel agar was supplemented with a 0.1 ml of yeast paste which increases egg laying by the flies. The flies were left in the cages overnight to ensure enough eggs have been laid on the surface of the embryo gel agar. The adult flies are subsequently removed from their cages. The embryo gel plates containing the laid eggs were left for 24 h after which the first instar larvae (L1) hatched. Upon emergence of the first instar larva, a soft pointed brush was used to pick up 25 L1 larvae, which were transferred to grape juice agar petri-dishes containing varying concentrations of the test chemicals. The L1 larvae were left to develop into adults over a period of 2 weeks.

The doses of different concentrations of BTEX that were used are as follows: benzene- 0.226 mM, 0.451 mM, 0.903 mM, 1.806 Mm, and 2.226 mM; toluene- 0.189 mM, 0.337 mM, 0.754 mM, 1.509 mM, 1.886 mM; ethylbenzene- 0.163 mM, 0.326 mM, 0.651 mM,1.303 mM, 1.6 mM; xylene- 0.08 mM, 0.160 mM, 0.320 mM, 0.640 mM, 1.290 mM, and 1.600 mM. These compounds were quickly added to 5 ml of grape juice agar and immediately covered. This is particularly important because the actual concentration of BTEX is expected to decrease as a function of time due to their volatile nature. Grape juice agar with and without DMSO added were used as control for this experiment. The experimental design made use of DMSO as the solvent.

Newly eclosed flies were counted on the 14th day through the 16th day at 25°C, after hatching, at which point it is presumed that all flies would have eclosed into adults under control conditions (Montgomery et al., 2014). Survival of flies in each vial group was recorded against concentration. Survival was defined as the ability of flies to eclose as adults before 16 days post-exposure to BTEX.

Initially for the DGRP lines, seven lines were randomly selected (RAL-832, RAL-38, RAL-41, RAL-882, RAL-509, RAL-859, and RAL-181) to test the suitability of the BTEX-assay for the entire panel of 200 lines. The methodology for harvesting L1 larvae in flies raised in cages as described above was adopted for all of the DGRP lines. From each of these lines a total of 75 L1 larvae were selected and raised in embryo gel plates containing a fixed concentration of p-xylene (1.068 mM) which is the LC_50_ value and were left to develop into adults over a period of 2 weeks. Only p-xylene was selected for the GWAS study because it resulted in the most reliable dose-response relationship in exposed flies. Due to the large number of animals utilized during the experiment, the assay was designed in batches and in triplicates of 25 L1 larvae per vial. To rule out batch effects in the panel, one strain (RAL-38) was repeated in all the batches.

### Immunohistochemistry

#### Effect of Benzene and p-Xylene Exposure on Cell Viability and Mitosis in Imaginal Disk Cells

We examined apoptosis and mitosis during wing imaginal disk development using Dcp-1 and Phospho-Histone H3 antibody staining, respectively. The cellular effect of benzene a known mutagen and p-xylene which produced the most amenable dose- response data were evaluated. Flies were fed with 1.068 mM each of p-xylene and benzene, for 3 days followed by the dissection of 3rd instar larva to harvest the wing disks. In controls, the larvae were fed with 0.1% DMSO (Nazir et al., 2003) in distilled water. Immunohistochemistry of third instar larval imaginal disks was done according to the methods described by Roberts (1998) and Sarkissian et al. (2014). Mouse anti-Death caspase-1- (Dcp-1) and Rabbit anti- Phospho-Histone H3 (PH3) were the primary antibodies (1 μL of primary antibody in 500 μL of block buffer). Fixation and staining were as in Stormo and Fox (2016). Images were obtained using a ZEISS ApoTome.2 fluorescent microscope by ZEISS Research Microscopy Solutions and imaging data was collected using the associated Zen 2 Pro software.

### Statistical Analysis

Statistical analyses for the results were obtained using SPSS 20.0 software. To be able to determine the statistical significance of the results, the data was analyzed by One-way ANOVA and the Tukey’s Honest Significant Difference (HSD) was used in the *Post hoc*. The differences between groups were considered significant at *p* > 0.05. The resulting data from the analysis were expressed as mean ± standard error of the larval emergence. Lethal concentration of the test chemicals were arrived at using Regression Probit Transformed Responses.

### Genome Wide Association Study (GWAS)

To identify candidate SNPs that contribute to variation in animal phenotypes, we submitted the least squares line means of the trait (eclosion of flies 16 days post exposure to 1.068 mM of p-xylene) to the DGRP analysis pipeline^2^, GWA was completed on 144 of the 200 lines assayed, based on the sequence data available within the pipeline at the time of analysis. The DGRP Freeze 2 Release 5.46 GWA analysis uses simple linear model ANOVAs on approximately 2.49 million SNPs using the model *Y* = μ + *M* + *g* + ε, where *Y* is the line means adjusted for *Wolbachia pipientis* infection and five major inversion polymorphisms (*In(2L)t*, *In(2R)NS*, *In(3R)Y*, *In(3R)P*, and *In(3R)Mo*) in the DGRP, μ is the overall population mean, *M* is the effect of DNA (the effect of the SNP) variant being tested, and *g* is a polygenic component with covariance between lines determined by their genomic relationship and ε is the error variance (Huang et al., 2014).

In addition, we performed GWA analysis for survival with p-xylene, calculated as the difference of line means between flies that were reared on control medium and those that were reared on p-xylene supplemented medium, using the same pipeline. We report the top associations with *P* < 10^–5^, based on quantile-quantile plots, which showed deviations of observed *p*-values from expected values at this threshold. The polymorphism identified at the threshold of *P* < 10^–5^ could possibly contain false positives, but has come to be recognized as a standard in DGRP studies. Pairwise linkage disequilibrium was assessed between polymorphic variants using the r^2^ parameterization (Huang et al., 2014) to help evaluate to what extent clustered SNPs segregate independently. We categorized the lines that had fewer eclosion on p-xylene medium “poor performer” and the rest into “good performer.”

Furthermore, the DGRP output provided information on site class for each SNP: SNPs more than 5,000 base pairs from any known gene were identified as such; SNPs in coding regions were identified as synonymous, non-synonymous, downstream, UTR-3-prime or intron SNP variants, as appropriate.

When a phenotype file is submitted on the DGRP2 pipeline, the phenotype is adjusted for the effects of *W. pipientis* infection and five major inversions (*In(2L)t, In(2R)NS, In(3R)K, In(3R)P*, and *In(3R)Mo*) (Mackay et al., 2012). Because this infection is known to affect various fitness traits in *D. melanogaster* (Fry et al., 2004), we tested the effect of the infection on p-xylene (1.068 mM) exposure. For *W. pipientis* infection adjustment we fit a linear model with the infection status and major inversion genotypes as covariates and the raw phenotypes as the response variable.

## Results

### Acute Toxicity of Adult Flies to BTEX

We first determined the LC_50_ (the concentration which resulted in 50% mortality) in adult *D. melanogaster* following 96 h of BTEX exposure (96 h LC_50_). We also extrapolated the LC_5_ and LC_95_ (the concentrations which resulted in 5% and 95% mortality, respectively). We determined this value by generating dose response curves for concentrations of each of the test chemicals (Supplementary Figure 1). The LC_50_ of benzene, p-xylene, Toluene, and m-xylene was 0.807 mM, 0.168 mM, 0.166 mM, and 0.288 mM, respectively. Thus, it can be inferred that toluene is the most toxic at 4.86, 1.73, and 1.01 times more toxic than benzene, m-xylene and p-xylene in *w*^1118^ flies, respectively (Table 1). Extrapolation of LC_5_, LC_50_, and LC_95_ value for ethylbenzene and o-xylene was not possible following 96 h exposure due to extreme mortality at all doses tested. These results highlight the impact of BTEX compounds on adult *D. melanogaster* survival.

**TABLE 1:** Toxicity profile of benzene, toluene, ethylbenzene, and xylene in adult *w*^1118^ mutant *Drosophila melanogaster* in 4 days renewal exposure.

Treatments	Number of animals exposed	LC_5_ C.L. (mM)	LC_50_ C.L. (mM)	LC_95_ C.L. (mM)	Slope ± S.E.	D.F.	T.F.
benzene	20	0.058	0.807	11.252	*Y* = 0.134 + 1.437X ± 0.324	5	4.86
p-xylene	20	0.053 (0.097–0.001)	0.168 (0.570–0.088)	0.528 (114.232–0.260)	*Y* = 2.559 + 3.298X ± 0.514	4	1.01
toluene	20	0.002 (0.019–0.000)	0.166 (0.347–0.00)	16.451 (4.229E + 32–1.984)	*Y* = 0.642 + 0.824 X ± 0.397	4	1
m-xylene	20	0.100 (0.162–0.022)	0.288 (0.652–0.184)	0.831 (9.224–0.445)	*Y* = 1.933 + 3.579X ± 0.519	4	1.73
o-xylene**	20	–	–	–	–	–	–
ethylbenzene**	20	–	–	–	–	–	–

C.L., confidence limit; L.C., lethal concentration; D.F., degree of freedom; T.F., toxicity factor. ** 100% mortality.

### Developmental Progression Analysis in Animals Exposed to BTEX

We next examined the impact of BTEX compounds on developmental progression. We did so by examining the ability of newly hatched larvae to progress into adulthood in the presence of BTEX compounds. Animals fed DMSO + m-xylene, benzene, toluene, or ethylbenzene all exhibited some additional lethality relative to DMSO alone, though not in a dose-dependent manner (Table 2). However, we did observe consistent dose-dependent reductions (*p* < 0.05) in survival of animals fed p-xylene and o-xylene (Table 2). Overall, our results highlight an impact of BTEX compounds on animal development, with two xylene family compounds showing the most consistently detrimental effects.

**TABLE 2:** Survival of *w*^1118^ larvae exposed to BTEX in grape juice concentrate and 100% grape juice.

Test chemical	Experimental group (mM)	Total number of larvae exposed per replicate	Meannumber eclosed ± SE
p-xylene	dH2O	25	21.5 ± 0.50
	DMSO	25	21 ± 1.00
	0.08	25	18.5 ± 0.50
	0.16	25	18.5 ± 1.50
	0.32	25	16 ± 0.00**
	0.64	25	14.5 ± 0.50**
	1.29	25	10.0 ± 0.0**
	1.6	25	2.0 ± 1.0**
o-xylene	dH2O	25	21.5 ± 0.50
	DMSO	25	21 ± 1.00
	0.16	25	17.5 ± 0.50
	0.32	25	17.0 ± 2.00
	0.64	25	13.0 ± 2.00**
	1.29	25	10.0 ± 0.00**
	1.6	25	9.0 ± 1.00**
benzene	dH2O	25	21.5 ± 0.50
	DMSO	25	21.0 ± 1.00
	0.226	25	21.5 ± 1.50
	0.451	25	20.0 ± 0.00
	0.903	25	18.0 ± 2.00
	1.806	25	17.0 ± 2.00
	2.226	25	17.5 ± 0.5
toluene	dH2O	25	21.5 ± 0.50
	DMSO	25	21.0 ± 1.00
	0.189	25	20.0 ± 1.00
	0.377	25	15.5 ± 0.50
	0.754	25	15.50 ± 2.50
	1.509	25	18.0 ± 0.00
	1.886	25	15.5 ± 0.50
ethylbenzene	dH2O	25	21.5 ± 0.50
	DMSO	25	21.0 ± 1.00
	0.163	25	19.0 ± 1.00
	0.326	25	18.5 ± 0.50
	0.651	25	18.0 ± 1.00
	1.303	25	18.0 ± 0.00
	1.6	25	17.5 ± 0.50
m-xylene	dH2O	25	21.0 ± 1.00
	DMSO	25	19.5 ± 0.50
	0.16	25	19.0 ± 2.00
	0.32	25	16.0 ± 0.00
	0.64	25	19.0 ± 0.00**
	1.29	25	17.0 ± 1.00
	1.6	25	21.0 ± 1.00

Statistical significance, treated versus control group: **(*p* < 0.05) Results are expressed as means ± standard error. *N* = 3.

### P-Xylene Exposure Increase Both Apoptosis and Mitosis in Imaginal Disks

**FIGURE 1:**
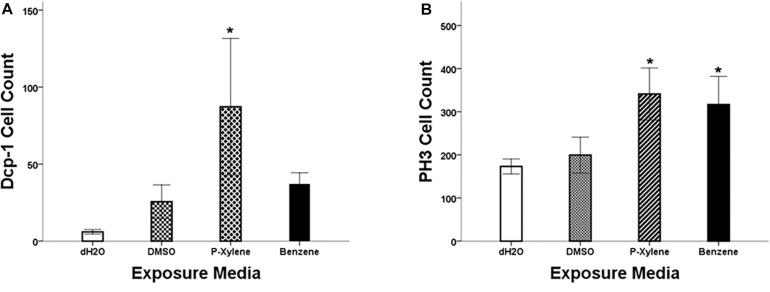
Quantitation of Dcp-1 and PH3 staining in wing imaginal disks following p-xylene and benzene feeding. **(A)** Summary of data showing activity of cleaved Dcp-1 in wing disks of *w*^1118^ 3rd instar larva. Data are represented by mean antibody-positive cell count ± SE (*N* = 8). **(B)**: Summary of data showing activity of PH3 in wing disks of *w*^1118^ 3rd instar larva. Data are represented by mean cell count ± SE (*N* = 6). Statistical significance, treated versus control group: * (*p* < 0.05).

**FIGURE 2:**
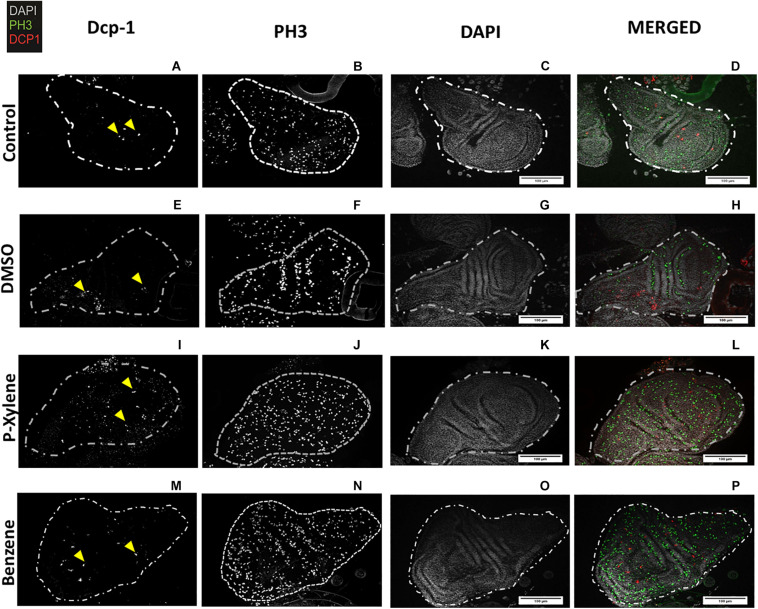
Visualization of Dcp-1 and PH3 staining in wing imaginal disks following p-xylene and benzene feeding. Wing disks of 3rd instar larvae immunostained for cleaved Dcp-1 (red), PH3 (green), and DAPI to visualize DNA (white). **(A–D)**. Representative images of cell division and apoptosis in wing disk of control flies. First instar larvae were treated with 4 μL of deionized water for 72 h as described in “Materials and Methods” section. **(E–H)** Representative images of cell death and apoptosis in DMSO treatment. First instar larvae were treated with 0.1% of DMSO for 72 h as described in “Materials and Methods” section. **(I–L)** Representative images of cell division and apoptosis in p-xylene. First instar larvae were treated with 1.068 mM of p-xylene for 72 h as described in “Materials and Methods” section. **(M–P)** First instar larvae were treated with 1.068 mM of benzene for 72 h as described in “Materials and Methods” section. Dashed lines indicate shape of *Drosophila melanogaster* wing disk. Scale bar = 100 μm.

To examine the impact of BTEX compounds on animal development at a cellular resolution, we examined rates of cell death (apoptosis) and cell division. We first examined apoptosis using Dcp-1 antibody staining. After feeding with 1.068 mM of p-xylene for 3 days, the number of dying cells on the wing disk epithelia was significantly increased by a magnitude of 3-fold when compared to controls. Dcp-1 activity was increased in benzene-fed animals, but it was not significant (Figures 1A, 2). In controls, the larvae were fed with 0.1% DMSO. Thus, an effect of the acute ingestion of p-xylene is a large increase in cell death in the cells of the *D. melanogaster* wing disk epithelium. In contrast, apoptosis in the benzene-treated wing disks was only increased up to about 2 times of that in controls. These data indicate that p-xylene markedly increases the number of apoptotic cells in the wing disk of *D. melanogaster* and reflects the general toxicity of this compound during larval development.

To determine if apoptosis caused by benzene and p-xylene treatment can result in signaling of the surrounding cells of dying epithelial cells to initiate tissue regeneration processes (Fox et al., 2020), wing imaginal disks were stained with an antibody against phospho-histone H3 (PH3), a specific marker of mitotic cells. In wing disks that were treated with 1.068 mM of p-xylene, there was a 2-fold increase in the number of the PH3-positive cells (Figures 1B, 2) which was significantly higher than those in control and DMSO. Similarly, animals that were fed with 1.068 mM of benzene for 3 days exhibited a significant 2-fold increase in the number of the PH3-positive cells in comparison with those in control and DMSO (Figures 1, 2). Together, these results suggest that exposure to p-xylene, and potentially benzene, kills *D. melanogaster* wing disk epithelial cells and stimulates compensatory cell division as part of an injury response.

### GWAS Screen for BTEX Susceptibility

Given our findings that *D. melanogaster* is a convenient model system to study the impact of BTEX compounds, we next leveraged the ability of this system to reveal genetic regulation of BTEX responses. We used the DGRP collection, a sequenced and inbred library of lines used in GWAS studies (Mackay et al., 2012; Mackay and Huang, 2018). We determined the LC_50_ and also extrapolated the LC_5_ and LC_95_ using dose response curves generated from concentrations of the test compound. Randomly selected DGRP flies exposed to Benzene, m-xylene, ethylbenzene and toluene did not respond in a dose dependent manner and therefore the extrapolation of their LC_50_ is not predictive of the effect of the toxins against the flies. However, for p-xylene, we saw a reproducible dose dependent effect, and chose an LC_50_ of 1.068 mM (Table 3) p-xylene to further use in GWAS analysis, using the eclosion assay described in sections “Larval Development Studies” and “Developmental Progression Analysis in Animals Exposed to BTEX.”

**TABLE 3:** Toxicity profile of BTEX against larvae of randomly selected DGRP lines.

Treatment	Number exposed per petri dish	LC_5_ C.L. (mM)	LC_50_ C.L. (mM)	LC_95_ C.L. (mM)	Slope ± S.E.	D.F.
benzene	25	6.03E + 8	0.00	0.00	*Y* = −0.490–0.131X ± 0.273	3
p-xylene	25	0.016 (0.082–0.00)	1.068 (100.928–0.553)	69.75 (2.060E + 13 –6.809)	*Y* = −0.026–0.906X ± 0.389	2
toluene	25	4.98E + 9	0.00	0.00	*Y* = −0.545–0.132X ± 0.273	3
m-xylene	25	0.00	1136.71	2.61E + 18	*Y* = −0.327 + 0.223 X ± 0.269	3
o-xylene	–	–	–	–	–	–
ethylbenzene	25	0.00	293.42	4.45E + 11	*Y* = −0.442 + 0.179X ± 0.278	3

*C.L., confidence limit; L.C., lethal concentration; D.F., degree of freedom.*

**FIGURE 3:**
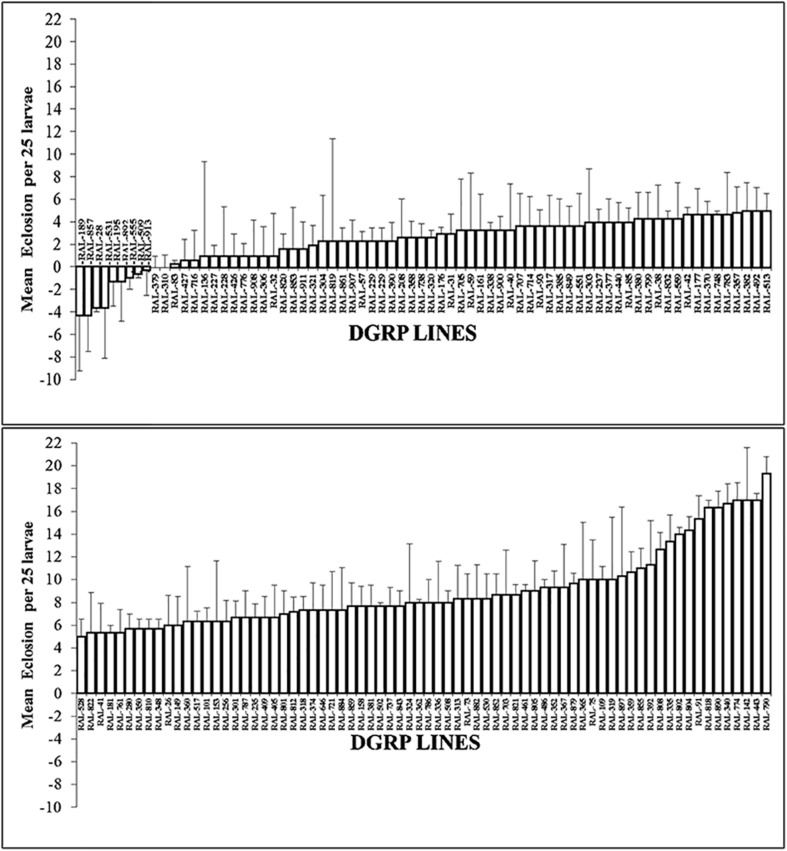
Variation in p-xylene eclosion rates among 144 DGRP lines.

To examine the suitability of the BTEX-assay for the DGRP lines and in order to arrive at a fixed dose for GWAS exposure, a panel of seven DGRP lines were selected (RAL-832, RAL-38, RAL-41, RAL-882, RAL-509, RAL-859, and RAL-181) at random. First instar larva were selected from these DGRP lines and allowed to feed on p-xylene treated plates for 24 h. Eclosion was recorded in each of the different concentrations. Only p-xylene was toxic in a dose dependent manner and showed variation in toxicity with the animals. To characterize natural variation in p-xylene response in 200 DGRP lines, a fixed dose of 1.068 mM p-xylene was selected. The animals were screened for survival post-p-xylene exposure, and we found extensive phenotypic and genetic variation in p-xylene exposure response across the tested DGRP panel (Figure 3). It was found that the lines fell into two distinct groups: one group of 56 lines with no survivors at all in the control (i.e., RAL line + DMSO) and p-xylene (i.e., RAL line + p-xylene) exposure, another group of 144 lines with survivors. The 144 lines were further grouped into 10 lines that showed resistance to p-xylene exposure, while the remaining 134 ranged from mildly susceptible to highly susceptible to p-xylene treatment and thus are sensitive. While lines such as RAL-189, RAL-857, RAL- 28, RAL-531, and RAL-195 did exceptionally well on a fixed concentration of p-xylene, RAL-790, RAL-443, RAL-142, RAL-774, and RAL-340 rarely eclosed 2 weeks post exposure in the presence of a fixed concentration of the test compound. We define performance as the mean difference of eclosion between control and of p-xylene treated media. Results for most lines were reproducible, with the exception of five highly variable lines (RAL-357, RAL-812, RAL-85, RAL-843, and RAL-362). Our results show that, for p-xylene, we identified differences in susceptibility within the DGRP collection. Importantly, it was observed that RAL-38 did not move from susceptible to resistant throughout the period of the experiment, suggesting there were minimal batch effects.

**FIGURE 4:**
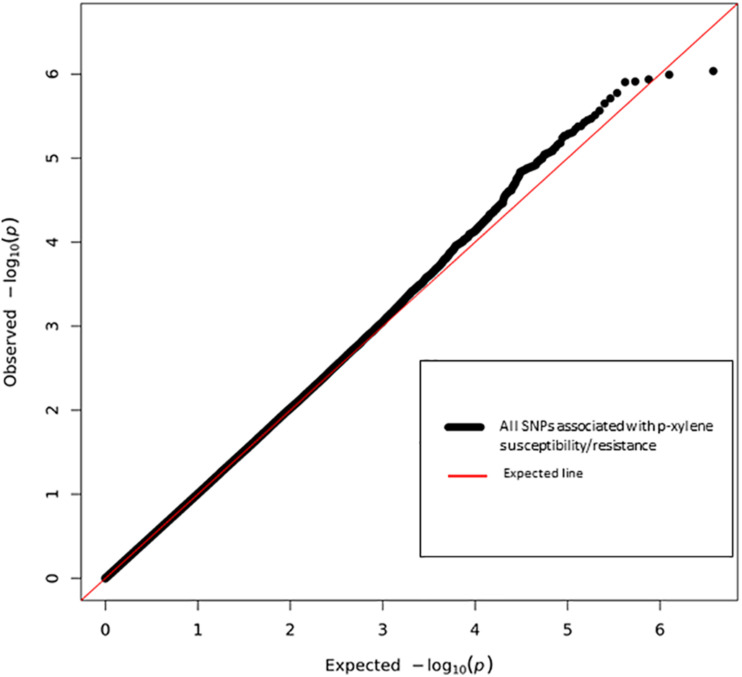
Quantile-Quantile plot of association analyses of p-xylene resistance and susceptibility among 144 DGRP lines. The red line indicates the expected and the black line the observed *p* values. Six top performing lines are highlighted.

**FIGURE 5:**
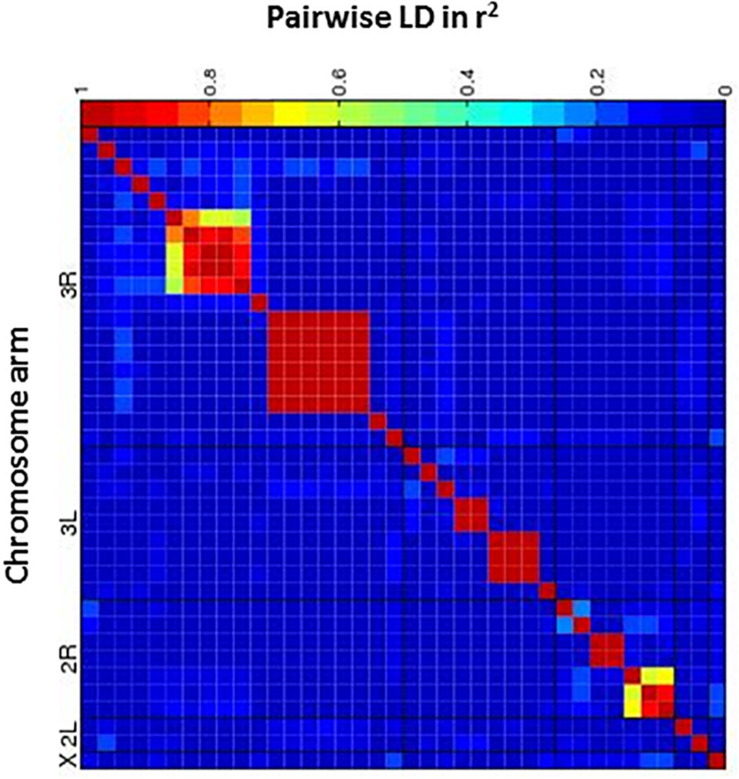
Heatmap of linkage disequilibrium (LD) values (R^2^) between candidate SNPs. The heat map depicts the degree of LD, r^2^, between variants. The five major chromosome arms are defined by the black lines. Red corresponds to complete LD and blue to absence of LD. A nominal *P* ≤ 10^– 5^ is indicated with a red line for each trait.

Next, we performed a case-control GWA analysis of the phenotype using SNPs from the DGRP freeze 1 sequencing data in order to identify genes that contain alleles conferring differences that correlate with p-xylene exposure susceptibility (Mackay et al., 2012). A total of 1,886,036 SNPs met the quality control thresholds and they were tested for association with p-xylene susceptibility using a linear mixed model (Supplementary Tables 1A,B). Using the Mackay lab GWAS protocol (Mackay et al., 2012), we identified SNPs that were significantly correlated with increased or decreased p-xylene susceptibility. A total of 38 SNPs were associated with both increased and decreased p-xylene susceptibility at *p* < 10^–5^, among which 34 were at *p* > 10^–6^, three (3) at *p* < 10^–5^, and one (1) at *p* > 10^–7^ (Supplementary Table 2 and Figure 4). The majority of the 38 SNPs were common variants, having a minor allele frequency of ≥5%. According to genomic site class, of the 38 SNPs, 10 were intronic, 4 fell in coding regions (one non-synonymous, four synonymous), three (3) occurred in the 3′ UTR, and nineteen (19) were intergenic or downstream of a gene. The heatmap of the pairwise Linkage Disequilibrium (LD) measurement generated by the call for the 38 SNPs reveals that the rate of LD decay is substantially lower on the X chromosome when compared to the autosomes (Figure 5).

Excluding the intergenic regions, the remaining SNPs implicated 16 genes, 14 of which have human homologs. In particular, these data highlight the importance of *chaff (cha)* with respect to p-xylene susceptibility: a total of 5 polymorphisms (5 SNPs) were found in the first intron of this gene, and they are associated with natural variation in p-xylene in this analysis. *chaff* is a known maternal effect lethal and female sterile gene (Lindsley and Zimm, 1992). Besides the *chaff* gene with 5 hits, three other genes had multiple hits: *toutatis* (*tou*), which is primarily involved in transcription factor binding activities and regulation of chromatin structure (Fauvargue et al., 2001), had three SNPs located on the gene; *defective proboscis extension response* (*dpr6*) gene had two SNPs and it is known to form complexes that specify synaptic connections between neurons and target cells (Carrillo et al., 2015); *phosphoglucomutase 2b* (*CG10202*) had two SNPs and it is a protein coding gene that is predicted to be involved in carbohydrate metabolic process. Overall, we mapped SNPs in sixteen (16) genes in the GWA analyses for exposure to p-xylene using a significant criterion of *p* < 10^–5^. The genes are *mdh2*, *pnt*, *tweek*, *CG13532*, *CG33970*, *CG10202*, *Irk1*, *DmsR*-1, *Meltrin*, *CG32112*, *Nckx30C*, *tou*, *dpr6*, *Dys*, *CR43451*, and *cha*. All but two of the genes namely *CR43451* and *CG13532* have human homologs (Supplementary Table 3). We note that the *cha* and *Dys* genes are involved in biosynthesis of the neurotransmitter acetylcholine and the modulation of chemical synaptic transmission, respectively. While many of the candidate genes have primary functions and activities to which they are associated, for *CG13532*, *CG32112*, and *CR43451*, the molecular functions and the biological processes in which they are involved are not known, suggesting our analysis may reveal novel gene functions. All genes found in our screen are novel candidates in p-xylene susceptibility and resistance (Supplementary Table 3).

## Discussion

In this study, our results show that, despite the volatility of most BTEX compounds, both larval and adult stage *D. melanogaster* can be used to study effects on developmental and acute toxicity. Our results reveal compound and dose-dependent effects on both adult animal survival and progression through larval and pupal development. Further, we find effects at the cellular level on both apoptosis and mitosis, and reveal genome regions that regulate BTEX susceptibility.

Toluene and p-xylene were extremely toxic to adult *D. melanogaster* in the adult toxicity assay. The data revealed an order of toxicity of toluene ≥ p-xylene > m-xylene > benzene. It must be noted that in all exposed groups, the toxicity of the BTEX compounds followed a dose-dependent response, except in o-xylene and ethylbenzene, where there was no clear response pattern for mortality. It is possible that toluene and p-xylene might be metabolized more slowly and thus may require a more prolonged exposure as compared to a rapidly metabolizing benzene and m-xylene (Avramov et al., 2013). On the other hand, it is also possible that the duration of exposure to the test chemicals could have favored the toxicity of toluene and p-xylene (Niaz et al., 2015). Toluene also promotes differential gene expression in *D. melanogaster* as described by Moskalev et al. (2014).

The acute effect of BTEX compounds in different organisms is well documented in the literature (Pumo et al., 2006; Gao et al., 2014; Camara-Lemarroy et al., 2015). In African earthworms (*Eudrilus eugeniae*), 96 h LC_50_ values for exposure to xylene, toluene, ethylbenzene and benzene were 0.011 mmol/kg, 0.014 mmol/kg, 0.013 mmol/kg, and 0.024 mmol/kg, respectively (Doherty et al., 2019). In Asian earthworms (*Perionyx excavatus*), toluene was acutely toxic with LC_50_ value of 0.5 μmol/cm^2^ after a 48 h exposure (An and Lee, 2008).

Results from our exposure of first instar larvae to benzene, ethylbenzene, o-, m-, p- xylene and toluene reveals that lower concentrations of the test substance has a milder effect on animal development when compared with the higher concentrations that resulted in developmental delays beyond 16 days post exposure. This effect was more significant in o-, m-, and p- xylene. It is possible that these toxicants act in a different way, other than direct effect on survival, to cause developmental delay in the exposed animals. Although the mechanism of the developmental toxicity of technical xylene components is unknown, it is possible that modulation of their metabolism may affect their toxicities. The response observed may be a result of environmental and genetic factors rather than from the toxicant themselves. Congruent to this study, a previous inhalation study in rats found that the three isomers of xylene produced developmental toxicity at concentrations between 500 and 2000 ppm (Ungvary et al., 1980; Saillenfait et al., 2003). In *D. melanogaster* in particular, exposure to benzene, xylene and toluene induced a delay in the number of flies that emerged into adult (Singh et al., 2009). In *Xenopus laevis*, treatment of tadpoles with p-xylene resulted in a significantly higher mortality, malformed tadpoles and developmental delay, in embryonic toxicity studies (Gao et al., 2016). In humans, there is also a relationship between high concentration of BTEX and neural tube defects in pregnant women (Lupo et al., 2011).

In addition to the developmental defects we observed at an organismal level, we also observed BTEX associated death (apoptosis) at the cellular level. Apoptosis is dependent on activation of caspases and is triggered during embryogenesis and normal tissue homeostasis in response to certain physiological changes. These changes are initiated by the activation of specific pathways, followed by changes in morphology such as nuclear and cytoplasmic condensation, cell shrinkage, increase or decrease in cellular ion concentration, DNA fragmentation and the release of a cellular component known as apoptotic bodies (Ai et al., 2011; Singh et al., 2011; Favaloro et al., 2012). Our study shows that the number of apoptotic cells was significantly increased in wing disks exposed to p-xylene compared to control media. Benzene, also induced an increase in apoptosis, but our results suggest it does not produce a significant effect on the number of dying cells, though we did observe an increased rate of mitosis in these animals. It is well documented that Reactive Oxygen Species (ROS) are generated during the metabolism of benzene and xylene (Singh et al., 2009) and availability of excessive free radicals is a culprit in cellular damage (Sarma et al., 2011). It has recently been found that xylene toxicity in human lymphocytes is stimulated through the generation of ROS (Salimi et al., 2017). These highly reactive radicals that are generated by benzene and p-xylene may have triggered a series of protein-protein interaction that then leads to an increase in membrane permeability of the cells. The ROS generated-effects may have moved from one cell to another through apoptotic signaling and thereby propagating there production in surrounding cells (Santabarbara-Ruiz et al., 2015). The results obtained could also be as a result of the induction of caspase-dependent cell death pathways mediated by mitochondria (Singh et al., 2011).

We observed increased cell division in developing tissues exposed to some BTEX compounds. Cell proliferation is a known mechanism by which a number of tissues in *D. melanogaster* respond to death of cells (Sun and Irvine, 2011; Yang et al., 2014). Administration of benzene in cultured porcine ovarian granulosa cells stimulates cell proliferation (Tarko et al., 2019). Similar to this study, benzene was confirmed to have the ability to induce chromosomal loss in wing primordial cells of *D. melanogaster* (Soós and Szabad, 2014). The increase in cell proliferation could be attributed to the activation of a cell death induced pathway that is required for regenerative growth in the wing disks of *D. melanogaster* in response to the increase in the number of dying cells (Bergantiños et al., 2010).

Our findings also represent a large-scale effort to identify genome wide associations that impact p-xylene exposure. One of the advantages of the DGRP collection is that it relies on freely occurring genetic variation. All strains in the DGRP panel were caught in the wild and inbred over twenty generations until isogenic (Mackay et al., 2012). This provides us with an opportunity to assess the magnitude of genotype by natural variation and their genetic basis, since complex behavior varies from individual to individual. Multiple studies have already utilized GWAS to generate novel interactors in complex traits such as aggression, stress response, and longevity, studies that were made possible by the intensive sequencing of all lines included in the DGRP (Shorter et al., 2015; Durham et al., 2014; Weber et al., 2012). It was then natural to leverage this tool to attempt a similar process in the field of toxicology. This type of study has been demonstrated with some range of toxicants including methylmercury, (Montgomery et al., 2014), radiation exposure (Vaisnav et al., 2014) and lead toxicity (Zhou et al., 2016).

This study identified a list of candidate genes that might play a role in modifying the phenotypic effect of p-xylene exposure. With the exception of two genes (*CR43451* and *CG13532*), all the top candidate genes have human orthologs (14/16) (Supplementary Table 3). This high level of conservation suggests that the findings in this study could be highly informative as human candidates in translational studies. Our p-xylene GWA analysis reveals candidate genes that were enriched for functions in nervous system development, neuromuscular synaptic transmission, membrane signaling factors, carbohydrate metabolism, imaginal wing disk development, transcription factor binding sites (TFBS) and apoptotic processes (Supplementary Table 3). The representation of these candidate genes shows that 3 of the top 16 candidates have human homologs that are linked to increased risk of certain human diseases such as Alzheimer’s disease, asthma, and chronic obstructive pulmonary disease (Meyer et al., 2010; Dey and Ray, 2018). There may be common pathways or mechanisms shared between these human diseases and exposure to p-xylene. There may well be an interaction between apoptotic genes and those responsible for development of the imaginal wing disks, these could explain the increase apoptosis and mitosis observed with p-xylene exposure in *D. melanogaster*.

Among the genes represented by the significant SNP hits, four contain multiple SNPs (*cha, tou, dpr6*, and *CG10202*). This is in concordance with Moskalev et al. (2014) whose study demonstrated that *cha* was downregulated in toluene treated flies. Future studies will be required to ascertain if truly the biological selectivity of these genes is representative enough or they are simply in linkage disequilibrium with causative variants found in other loci.

Two SNPs (3L_10034780_SNP and 3L_10034781_SNP) both downstream, mapped to the gene *dpr6*, are significantly associated with p-xylene resistance (*p* = 1.94 × 10^–6^ and 2.24 × 10^–6^). This result is particularly very interesting because a different GWAS study had mapped a SNP to the gene *Dpr6* that was associated with variation in *Providencia rettgeri* load in flies reared on different glucose diet (Unckless et al., 2015). *Dpr6* belongs to a family of genes thought to be involved in synapse organization and that localizes to the neuron projection membrane, including gustatory perception of food, and contains an immunoglobulin domain that may be involved in cell-cell recognition. The gene is expressed in the medulla and ventral nerve cord (Nakamura et al., 2002; Carrillo et al., 2015). This genetic correlation could mean that resistance to p-xylene is linked in some way to the metabolic uptake and conversion of glucose, but a full characterization of this mechanism will require future study.

The *cha* gene is responsible for the synthesis of the neurotransmitter acetylcholine (ACh) and this is typical of the cholinergic neurons present in the peripheral and central nervous system. In a reversible reaction *cha* catalyzes synthesis of ACh from acetyl-CoA and choline, ACh then stimulates muscle contraction in the central nervous system and learning in the central nervous system (Cai et al., 2004). Due to the high number of SNPs that were mapped to this gene relative to all other top annotations, p-xylene could have a major modulatory effect on the regulation of the *cha* gene and by extension a defect in synaptic transmission. This was demonstrated in zebrafish where a missense mutation in the homolog of the *cha* gene *chata* shows a strong reduction in embryo motility (Joshi et al., 2018).

Although it has been reported that xylene can induce DNA damage and candidate gene studies have linked xylene with chronic myeloid leukemia and cancer (Lim et al., 2016), none of our top 16 candidate genes from the 38 significant hits are associated with DNA damage. This could be due to the fact that the mode of toxic action of p-xylene in *in vitro* and in whole animal conditions is quite different from what is observed at the genome-wide level studies.

In summary, our study reveals 38 SNPs associated with p-xylene resistance and susceptibility in *D. melanogaster* developmental and toxicity experiments involving benzene, toluene, ethylbenzene, p-xylene, m-xylene, and o-xylene. The study also suggests that benzene and p-xylene are capable of eliciting apoptotic and cell proliferative responses in imaginal wing disk of *D. melanogaster*. The GWA analyses in this study has demonstrated the strength of the DGRP in revealing a highly polygenic genetic architecture that underlies variation in susceptibility to p-xylene toxicity, which may give rise to subtle variations in neuromuscular synaptic transmission during early development. The study further reveals genes 15 genes, some of which are associated with p-xylene exposure and whose human homologs have been linked with increased risk of certain human diseases. Future functional studies involving p-xylene exposure should consider looking at the absence or presence of these genetic variants using existing mutant or RNAi strains. Such studies could lead to future work involving critical gene expression or proteomic responses to BTEX compounds. Additionally, our findings here can serve as a guide for future population-based studies in humans.

## Data Availability Statement

The datasets presented in this study can be found in online repositories. The names of the repository/repositories and accession number(s) can be found in the article/ Supplementary Material.

## Author Contributions

DF, AO, and TA contributed to the conception and design of the study. TA performed the experiments, data analysis, and wrote the first draft of the manuscript. All authors contributed to manuscript revision, read, and approved the submitted version.

## Conflict of Interest

The authors declare that the research was conducted in the absence of any commercial or financial relationships that could be construed as a potential conflict of interest.

## Footnotes

1 http://archonscientific.com/recipes/

2 http://dgrp.gnets.ncsu.edu/
