# Supplementary material for: Toxicological Study and Genetic Basis of BTEX Susceptibility in Drosophila melanogaster
Source: Front Genet. 2020 Oct 15;11:594179. doi: 10.3389/fgene.2020.594179 (PMC7593870; doi:10.3389/fgene.2020.594179)
Supplement: Supplementary file 1 [file Data_Sheet_1.ZIP › SUPPLEMENTAL MATERIAL PRESENTATION/Supplementary Figure 1.docx]

**PROBIT TRANSFORMED RESPONSES FOR ADULT *w^1118^***


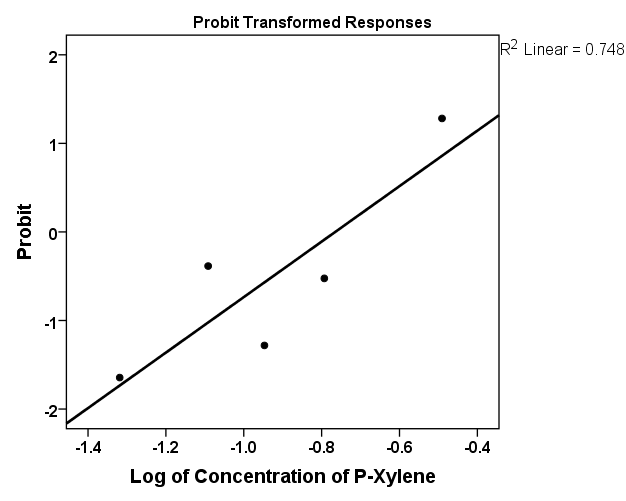


Probit transformed curve of p-xylene against adult flies


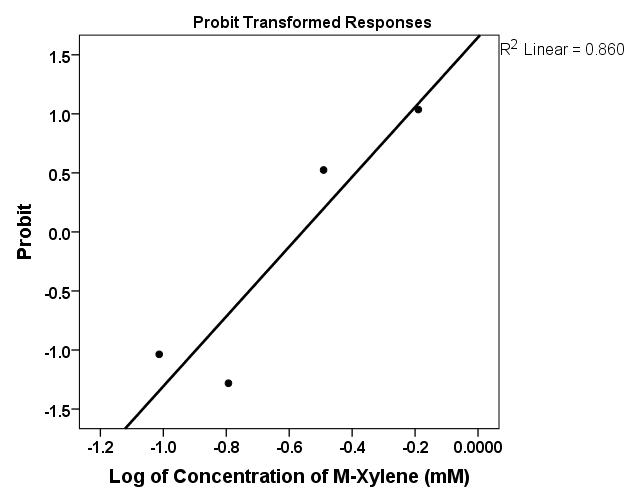


Probit transformed curve of m-xylene against adult flies


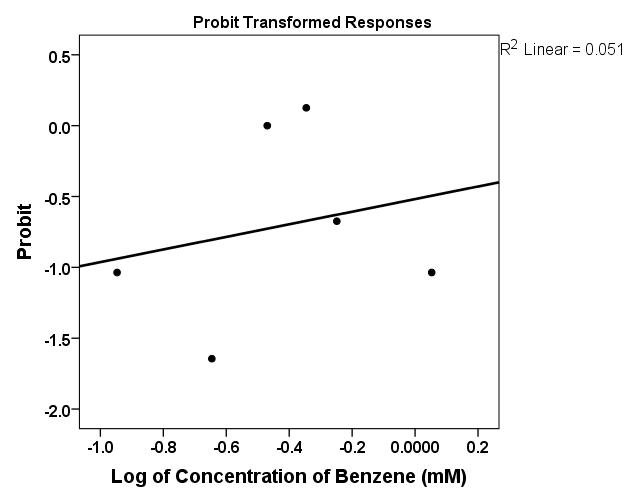


Probit transformed curve of benzene against adult flies


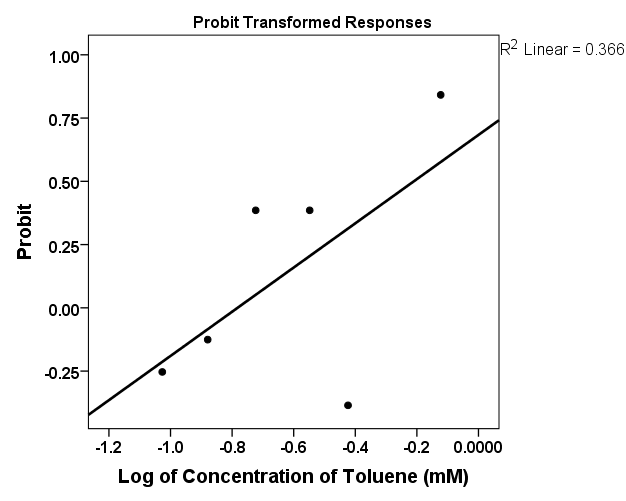


Probit transformed curve of toluene against adult flies

**PROBIT TRANSFORMED RESPONSES FOR LARVAE OF DGRP LINES**


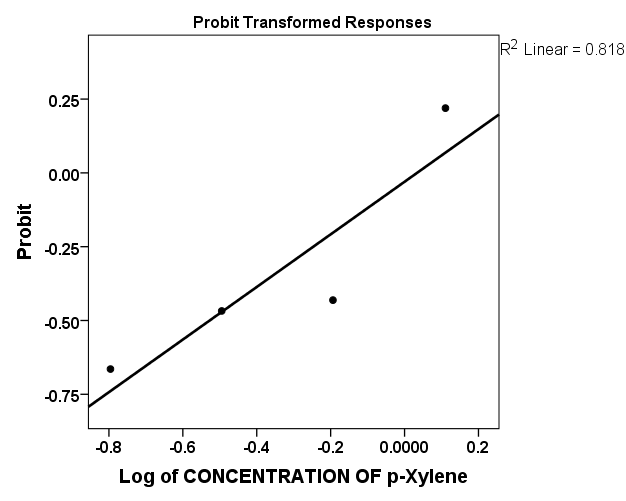


Probit transformed curve of p-xylene against DGRP lines


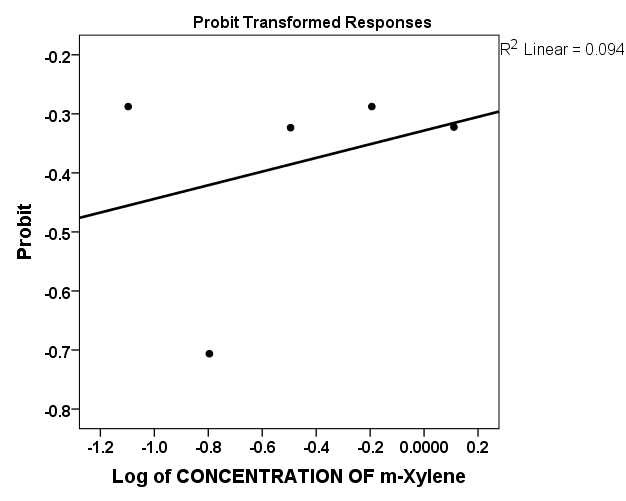


Probit transformed curve of m-xylene against DGRP lines


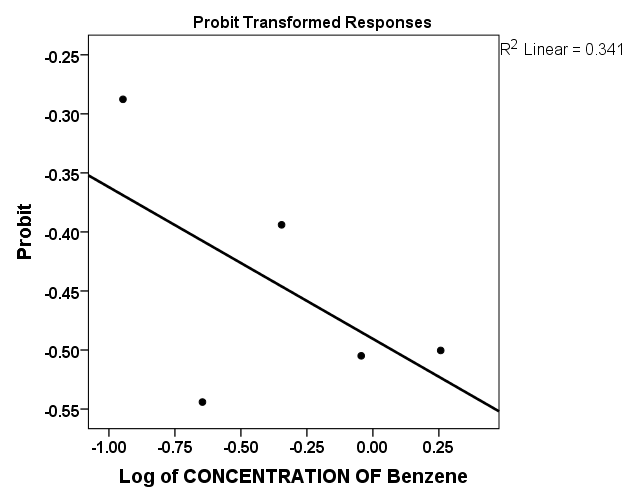


Probit transformed curve of benzene against DGRP lines


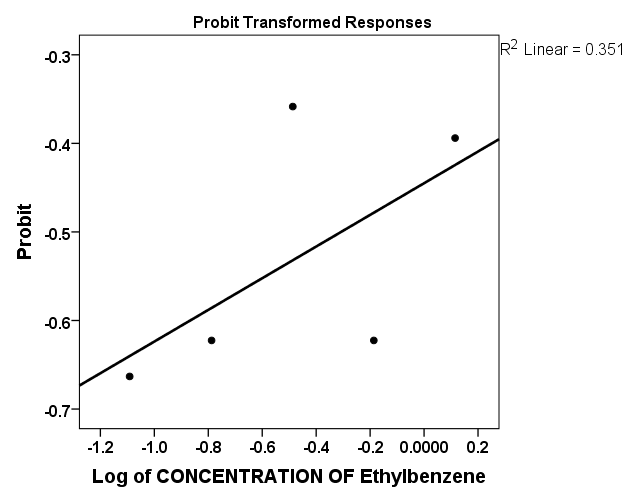


Probit transformed curve of ethylbenzene against DGRP lines


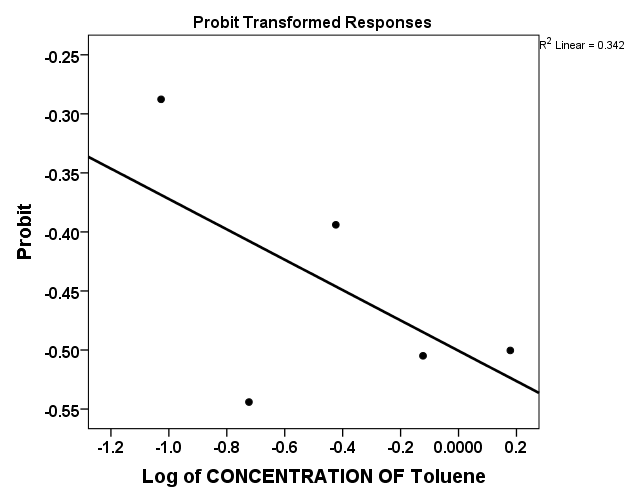


Probit transformed curve of toluene against DGRP lines
